# Supplementary material for: Improved Electrochemical Hydrogen Peroxide Detection Using a Nickel(II) Phthalimide-Substituted Porphyrazine Combined with Various Carbon Nanomaterials
Source: Nanomaterials (Basel). 2023 Feb 25;13(5):862. doi: 10.3390/nano13050862 (PMC10005363; doi:10.3390/nano13050862)
Supplement: Supplementary file 1 [file nanomaterials-13-00862-s001.zip › nanomaterials-2221375-supplementary.pdf]

Supplementary Materials

Improved Electrochemical Hydrogen Peroxide Detection Using a Nickel(II) Phthalimide-Substituted Porphyrazine Combined with Various Carbon Nanomaterials

Amanda Leda [1], Mina Hassani [2], Tomasz Rebis [1,*], Michal Falkowski [2,*], Jaroslaw Piskorz [3], Dariusz T. Mlynarczyk [4], Peter McNeice [5] and Grzegorz Milczarek [1]

[1] Institute of Chemistry and Technical Electrochemistry, Faculty of Chemical Technology, Poznan University of Technology, Berdychowo 4, 60-965 Poznan, Poland
[2] Department of Medicinal Chemistry, Collegium Medicum in Bydgoszcz, Faculty of Pharmacy, Nicolaus Copernicus University in Torun, Dr. A. Jurasza 2, 85-089 Bydgoszcz, Poland
[3] Chair and Department of Inorganic and Analytical Chemistry, Poznan University of Medical Sciences, Rokietnicka 3, 60-806 Poznan, Poland
[4] Chair and Department of Chemical Technology of Drugs, Poznan University of Medical Sciences, Grunwaldzka 6, 60-780 Poznan, Poland
[5] Faculty of Science and Engineering, Synthetic Organic Chemistry—Stratingh Institute of Chemistry and Chemical Building Blocks Consortium (CBBC), Nijenborgh 4, 9747 AG Groningen, The Netherlands
* Correspondence: tomasz.rebis@put.poznan.pl (T.R.); m.falkowski@cm.umk.pl (M.F.)

Table of Contents

1. General procedures..3
2. HPLC purity of porphyrazine 3..3
3. NMR study..8
Figure S1. ^{1}H and (^{13}C) chemical shift values [ppm] of **Pz3** and key correlations observed in NMR spectra.
Table S1. ^{1}H and ^{13}C NMR data obtained for **Pz3** including key correlations determined from ^{1}H-^{13}C HSQC and ^{1}H-^{13}C HMBC spectra.
Figure S2. ^{1}H NMR spectrum of **Pz3** (400 MHz, chloroform-d_1, 298 K).
Figure S3. ^{13}C NMR spectrum of **Pz3** (100 MHz, chloroform-d_1, 298 K).
4. Photochemical study..10
Table S2. UV–VIS absorption maxima (λ_{Abs}) and logarithms of molar absorption coefficients (logε) of **Pz3** in selected organic solvents.
5. Electrochemical study..10
Figure S4. Electrochemical reduction of GC/GO to GC/rGO in phosphate buffer
Figure S5. Chronoamperometric responses recorded at (A) GC/SWCNTs/**Pz3** and (B) GC/rGO/**Pz3**

1. General procedures

All reactions were conducted under argon atmosphere in glassware dried at 140 °C for at least 4 hours. All solvents were rotary evaporated at or below 60 °C under reduced pressure. The reported reaction temperatures refer to temperatures displayed by Radleys® Heat-On. Dry flash column chromatography was carried out on Merck silica gel 60, particle size of 40–63 μm and Aluminium oxide 90 active neutral (activity stage I) for column chromatography, 0.063-0.200 mm (Merck KGaA, Darmstadt, Germany). Thin-layer chromatography (TLC) was performed on silica gel Merck Kieselgel 60 F254 plates and visualized with UV light (λ_{max} = 254 or 365 nm). UV–VIS spectra were recorded on a Hitachi UV–VIS U-1900 spectrophotometer. The NMR spectra were acquired at 298 K on a full-fledged, two-channel 400 MHz Bruker AvanceCore NMR spectrometer. Chemical shifts (δ) are quoted in parts per million (ppm) and are referenced to a residual solvent peak – $CDCl_3$ (^{1}H NMR – 7.27 ppm; ^{13}C NMR – 77.00 ppm). Coupling constants *(J)* are quoted in hertz (Hz) and are rounded to the nearest halves or whole numbers. The abbreviations dd and t refer to doublet of doublets and triplet, respectively. ^{1}H and ^{13}C resonances were unambiguously assigned based on ^{1}H, ^{13}C, ^{1}H–^{1}H COSY, ^{1}H–^{13}C HSQC, and ^{1}H–^{13}C HMBC spectra. Mass spectra (MALDI-TOF) were carried out by the Wielkopolska Center for Advanced Technologies at the Adam Mickiewicz University in Poznan. All solvents and reagents were of reagent-grade quality, purchased from commercial suppliers, and used without further purification unless otherwise stated.

2. HPLC purity

The purity of macrocycle **Pz3** was determined by HPLC analysis using an Agilent 1200 instrument equipped with UV-DAD detector. The chromatographic separation was obtained on an octadecylsilane-coated column, 150 mm × 4.6 mm, 5 μm (Eclipse XDB-C18, Agilent), using gradient elution conditions at a flow rate of 1.0 mL/min. Band dispersion, and additional peaks from aggregates significantly hampered HPLC analysis. The best conditions for each compound are shown below. Peaks of minor components were detected, but the impurity content never exceeded 5% of the total signal intensity.

Configuration 1

	phase		
time [min]	methanol	dichloro-	water
0	95	0	5
3	95	0	5
4	50	50	0
15	50	50	0

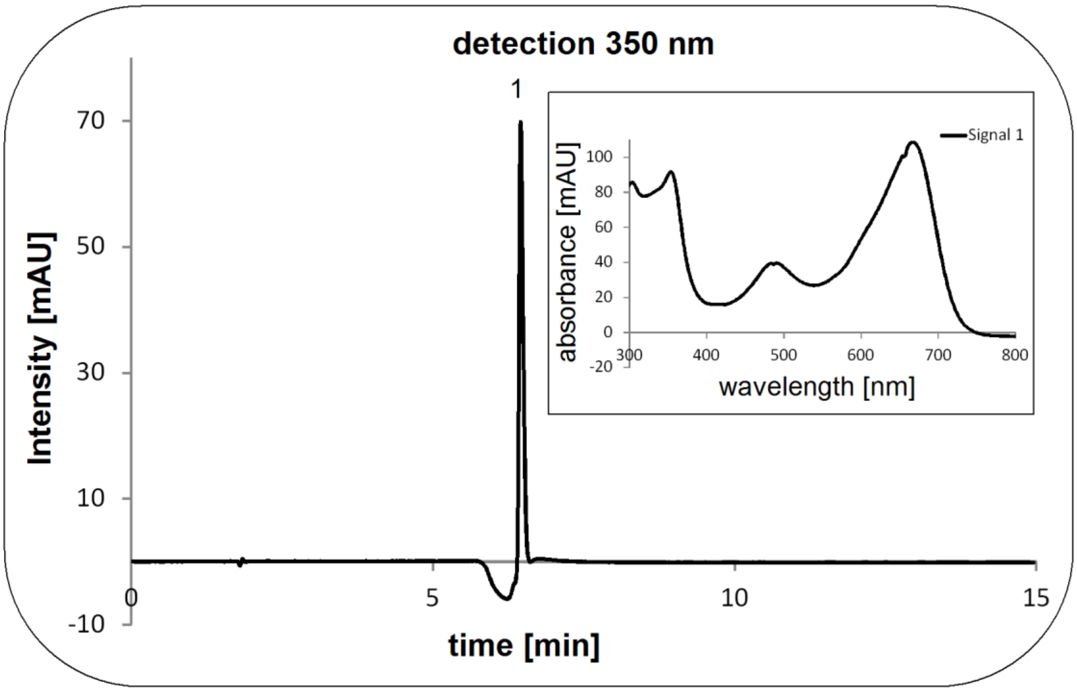

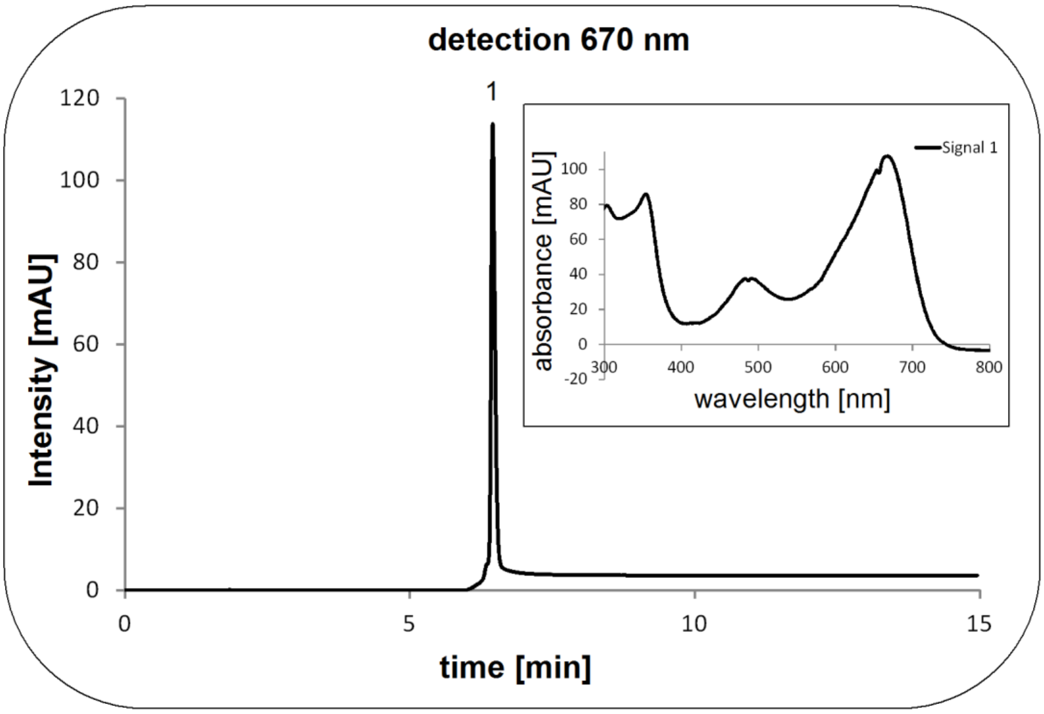

results			
signal	retention time [min]	area	purity [%]
detection 350 nm			
1	6.5	378.9	100.0
detection 670 nm			
1	6.5	584.5	100.0

Configuration 2

phase				
time [min]	acetonitrile	methanol	water	tetrahydrofu-
0	60	25	10	5
3	60	25	10	5
4	10	5	0	85
15	10	5	0	85

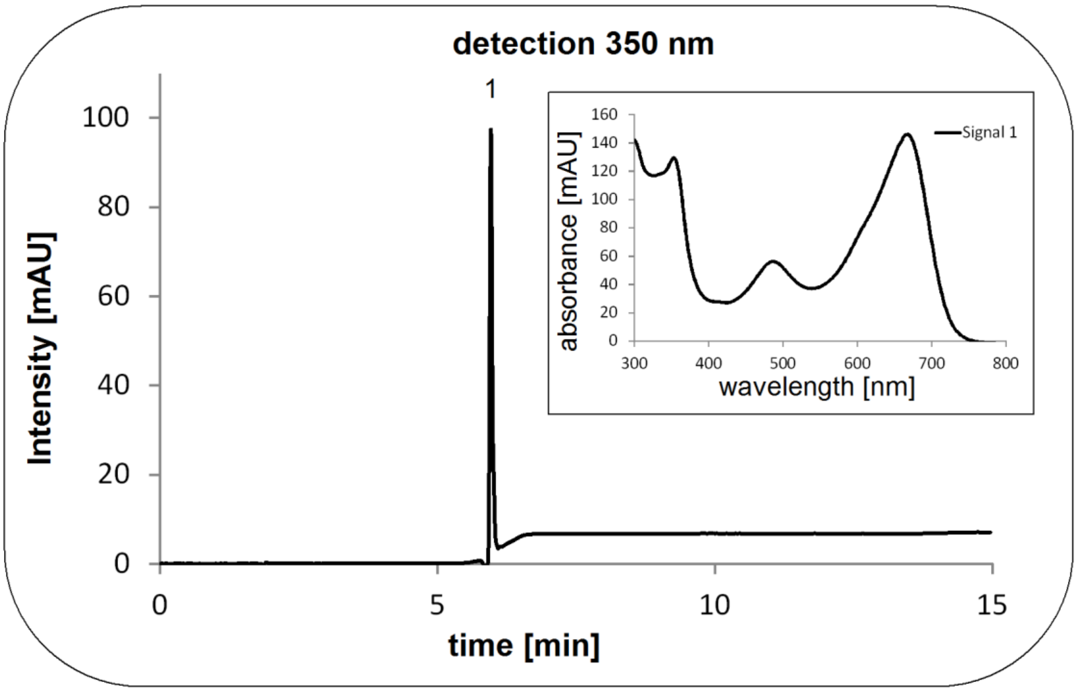

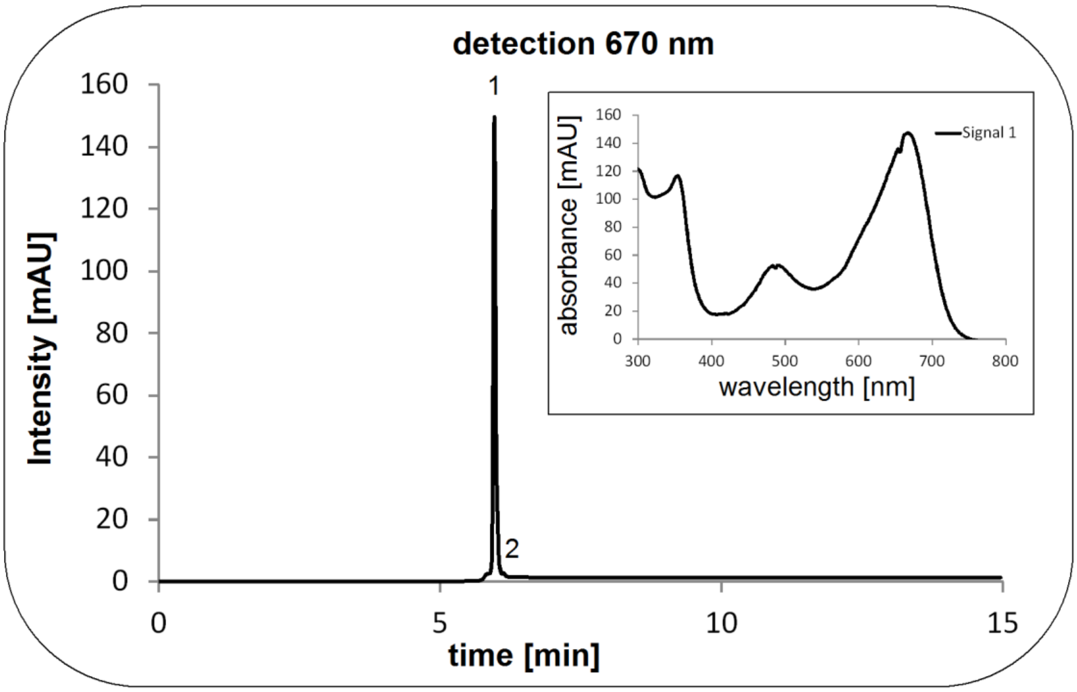

results			
signal	retention time [min]	area	purity [%]
detection 350 nm			
1	6.0	334.7	100.0
detection 670 nm			
1	6.0	503.2	98.5
2	6.1	7.7	1.5

Configuration 3

phase				
time [min]	methanol	water	dichloro-	tetrahydrofu-
0	80	10	5	5
3	80	10	5	5
4	20	0	40	40
15	20	0	40	40

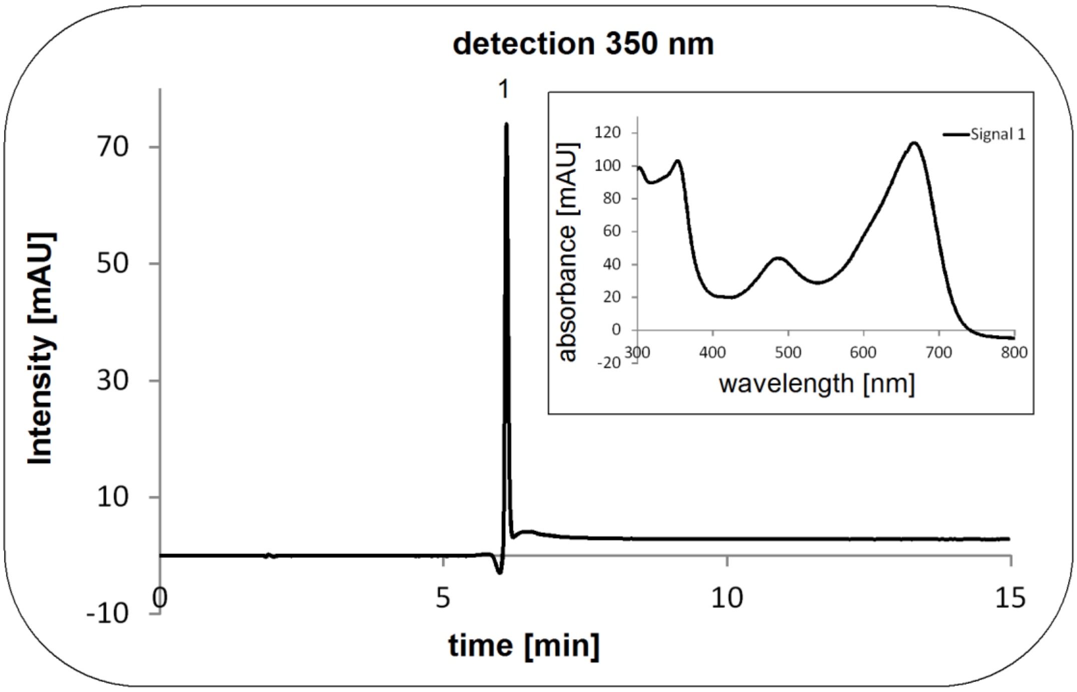

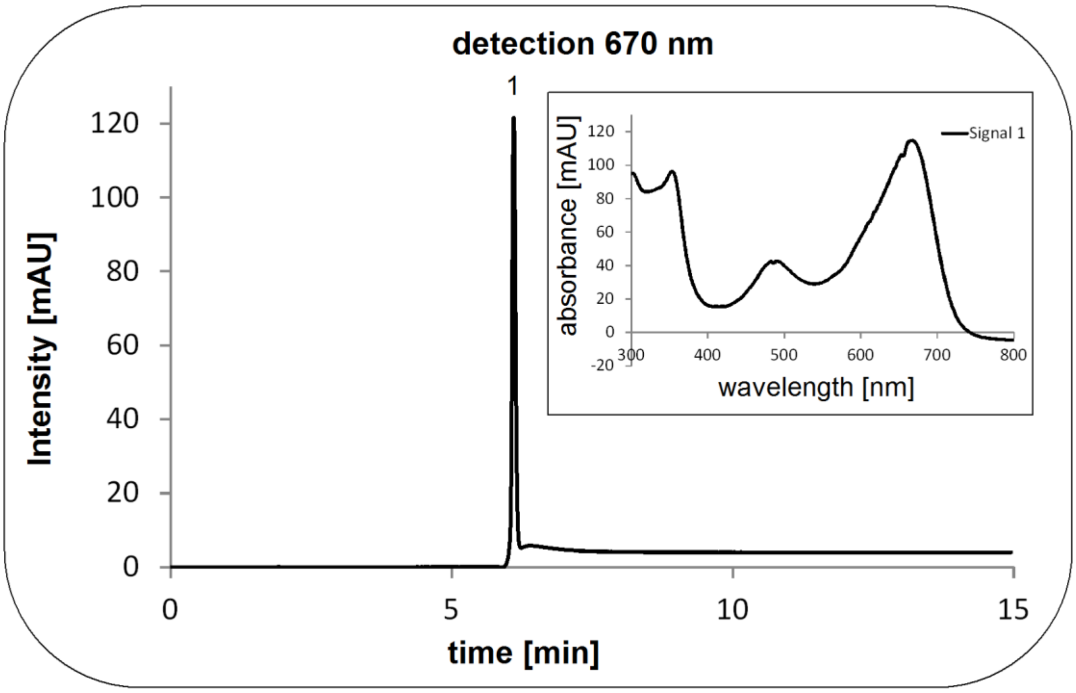

results			
signal	retention time [min]	area	purity [%]
detection 350 nm			
1	6.1	321.2	100.0
detection 670 nm			
1	6.1	507.1	100.0

3. NMR study

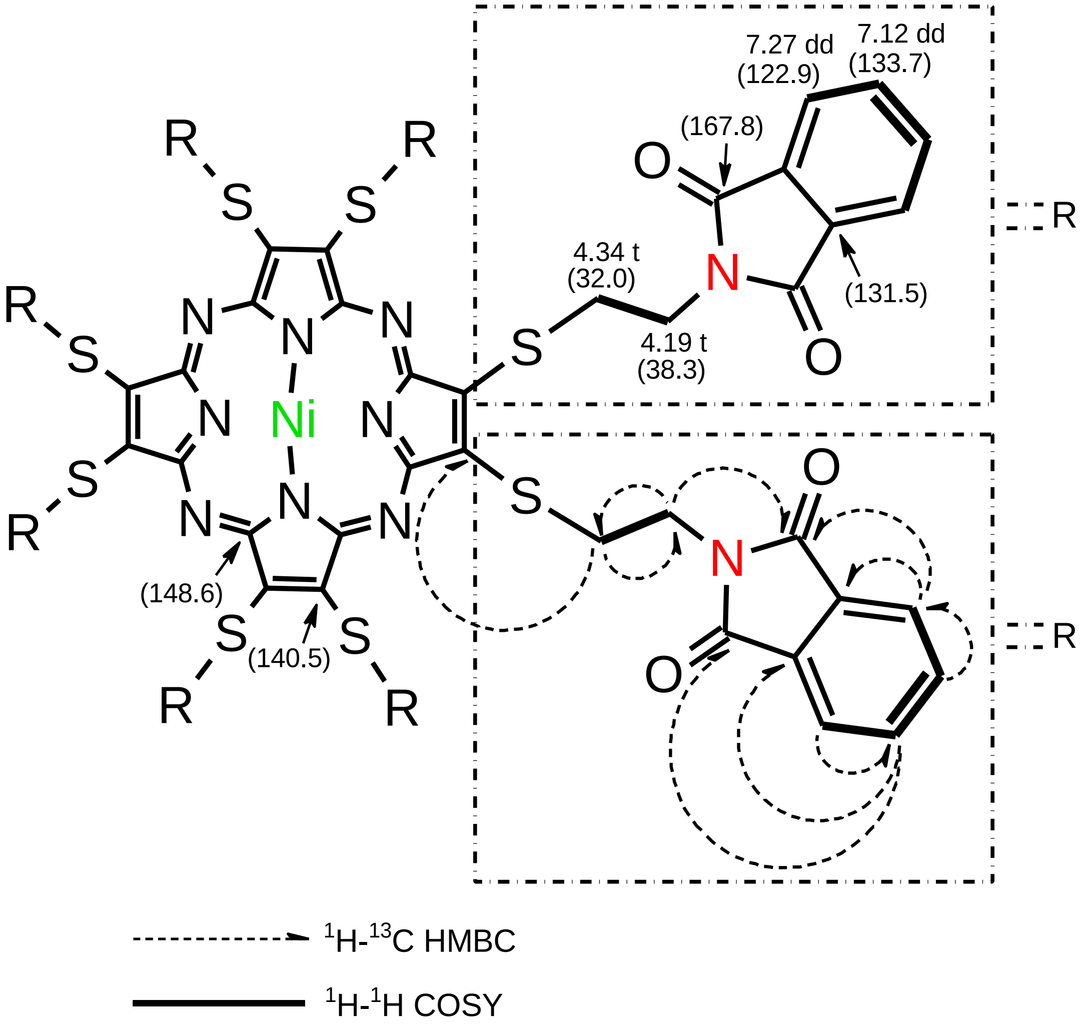

Figure S1. NMR data of **Pz3**: ^{1}H and ^{13}C chemical shift values [ppm] and key correlations observed in NMR spectra. Bold lines: ^{1}H–^{1}H COSY; Dashed arrows: ^{1}H–^{13}C HMBC.

Table S1. ^{1}H and ^{13}C NMR data obtained for **Pz3** including key correlations determined from ^{1}H-^{13}C HSQC and ^{1}H-^{13}C HMBC spectra.

δ_H (ppm)	Multiplicity (J_{H-H} in Hz)	^{1}H–^{13}C HSQC δ_C (ppm)	^{1}H–^{13}C HMBC δ_C (ppm)		
7.27	dd (5.5, 3.0)	122.9	167.8	133.7	131.5
7.12	dd (5.5, 3.0)	133.7	167.8	131.5	122.9
4.34	t (6.5)	32.0	140.5	38.3	
4.19	t (6.5)	38.3	167.8	32.0	
Other quaternary carbon atoms: 148.6 (ppm)					

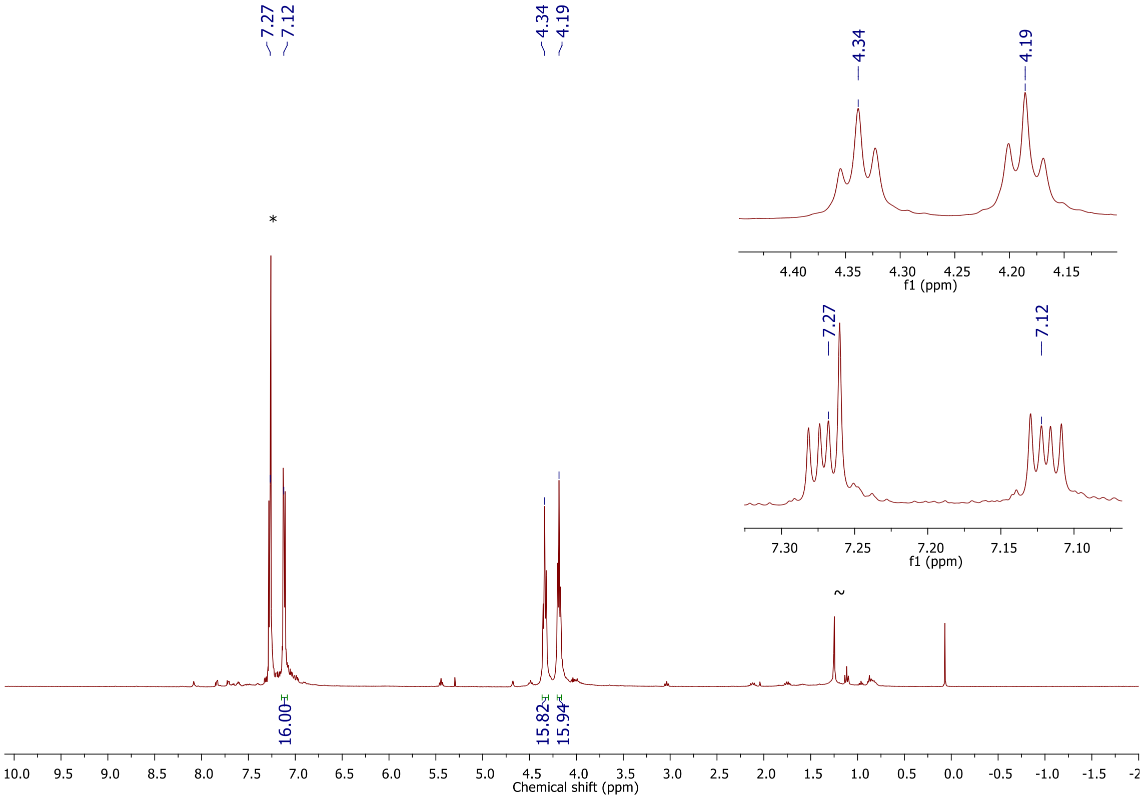

Figure S2. ^{1}H NMR spectrum of **Pz3** (400 MHz, chloroform-d_1, 298 K). The symbols * and ~ indicate chloroform-d_1 and water residual peaks, respectively.

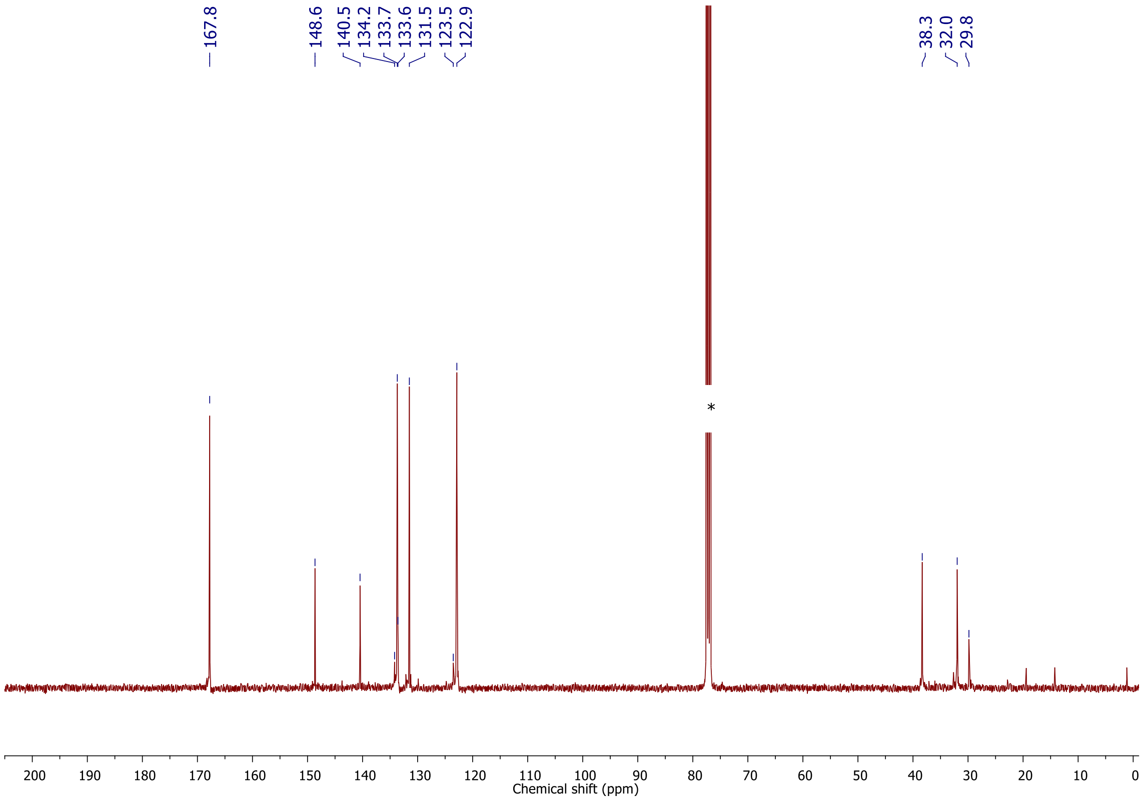

Figure S3. ^{13}C NMR spectrum recorded for **Pz3** (100 MHz, chloroform-d_1, 298 K). The symbol * indicates chloroform-d_1 residual peak.

4. Photochemical study

Table S2. UV–VIS absorption maxima (λ_{Abs}) and logarithms of molar absorption coefficients (logε) of **Pz3** in selected organic solvents.

Solvent	λ_{1Abs} (log ε)	λ_{2Abs} (log ε)	λ_{3Abs} (log ε)
Dichloromethane	303 (4.54)	352 (4.56)	667 (4.58)
N,*N*-Dimethylformamide	304 (4.52)	352 (4.52)	668 (4.57)
Dimethylsulfoxide	303 (4.56)	352 (4.55)	667 (4.58)

5. Electrochemical study

Multi-walled carbon nanotubes (MWCNTs, with an average diameter of 10 nm and an average length of 1.5 μm) were delivered by Metrohm DropSens (Oviedo, Spain). Single-walled carbon nanotubes (SWCNTs, with an average diameter of 2 nm and an average length of 3 μm), dichloromethane (DCM, puriss. p.a., ≥99.9%), N,N-dimethylformamide (anhydrous 99.8%), and hydrogen peroxide stock solution 30 wt. % were provided by Merck (Darmstadt, Germany). Graphene oxide (GO, water dispersion at a concentration of 4 mg/mL) for the preparation of reduced graphene oxide (rGO) were purchased from Nanopoz (Poznan, Poland). Glucose, fructose, lactose, maltose, and saccharose were delivered by Warchem (Warszawa, Poland). Caffeine was delivered by Fluka (Buchs, Switzerland). Monosodium and disodium phosphates for the preparation of phosphate buffer (PB, pH 7.4) were supplied by POCH (Gliwice, Poland). Sodium chloride, potassium hexacyanoferrate(II) trihydrate, and potassium hexacyanoferrate(III) were provided also by POCH (Gliwice, Poland). All of these compounds were reagent grade, thus they did not need to be purified further.

All of the electrochemical experiments were performed using an EmStat4S electrochemical analyzer (Eindhoven, Netherlands). A three-electrode system was used, with a reference electrode of Ag/AgCl (3 M KCl) and a counter electrode of a platinum wire. The working electrode was a glassy carbon electrode (GC) with a diameter of 3 mm (BASi, USA).

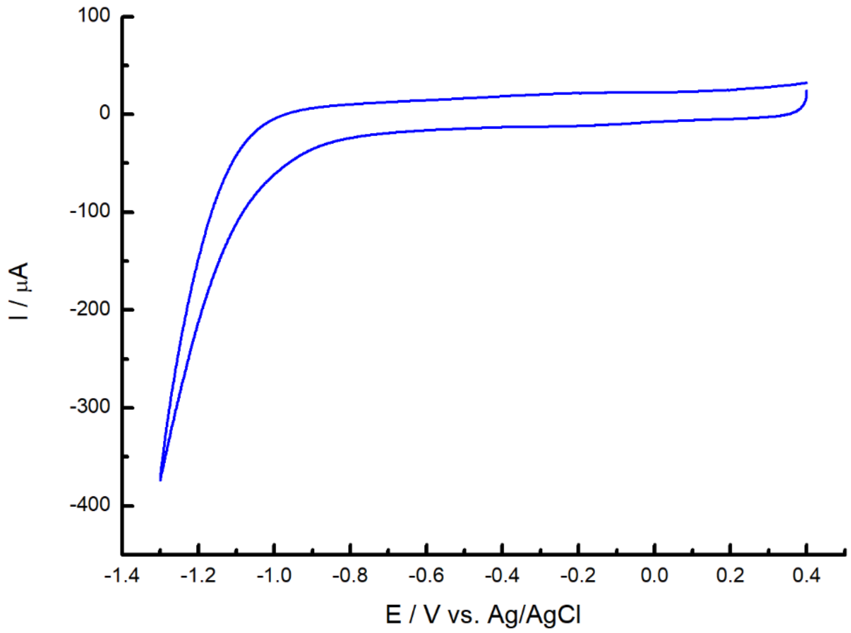

Figure S4. Electrochemical reduction of GC/GO to GC/rGO in phosphate buffer (PB, pH 7.4) in the potential range from 0.4 to −1.3 V (scanning rate 50 mV s^{-1}).

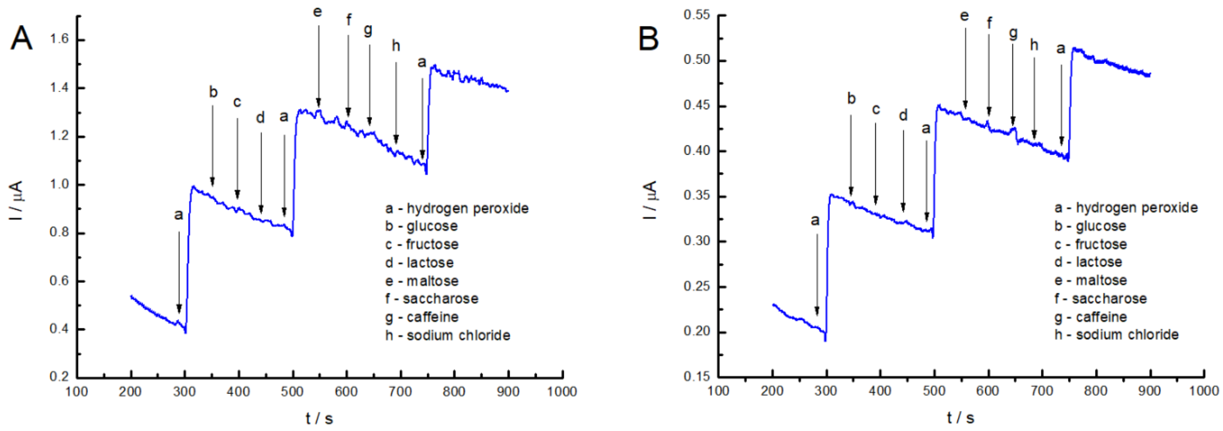

Figure S5. Chronoamperometric responses recorded at (A) GC/SWCNTs/**Pz3** and (B) GC/rGO/**Pz3** sensor upon addition of 100 µM H_2O_2 and selected interferents. The applied potential was +0.6 V. The supporting electrolyte was PB (pH 7.4).
